# From the Editor's Desk

**DOI:** 10.4103/0974-1208.57221

**Published:** 2009

**Authors:** Kamini A Rao

**Affiliations:** Editor-in-Chief, Journal of Human Reproductive Sciences E-mail: kambacc@vsnl.com

Assisted reproduction is one of the fastest growing areas of medicine having expanded far beyond the imaginations of those who pioneered the techniques that led to the birth of Louise Brown. Thirty odd years after her birth, infertility treatment has improved substantially. Not only has the process of uniting egg and sperm outside the body become a commonly practiced procedure, assisted conception treatments are proving to be more efficient and cost-effective than most traditional remedies. The availability of cryopreservation technology has extended the scope of infertility treatment and proved to be a boon for patients. Assisted hatching, preimplantation genetic diagnosis, ovarian tissue freezing, stem cell technology,… the treatment options are mind boggling. This requires the healthcare provider to be well equipped with the present scenario and JHRS attempts to do just that. Thanks to our insistence on the quality of submitted manuscripts and the journal's careful review process, each issue of the JHRS contains articles of exceptionally high standards. Once again we have selected a number of articles that definitely warrant mention in the editorial.

Genetic variation and environmental factors contribute to susceptibility to spermatogenic impairment in humans. Kiran Singh and Rajiva Raman have attempted to find out the possible association between A386G (T54A) polymorphism of the autosomal gene, *DAZL* and idiopathic male infertility in patients from North India. To date, there has been no clear insight into the functional significance of the chromosome-encoded *DAZ* gene family in spermatogenesis. Kiran Singh and Rajiva Raman's results illustrate the rarity of this mutation.

Dr. Sonal Panchal has presented her study, which shows that 3D and 3D Power Doppler when used with 2D US and color Doppler for pre-hCG follicular assessment would definitely improve pregnancy rates in IUI cycles. Although still larger studies are needed to establish more precise values for follicular VI and FI, the results are definitely promising.

Dissanayake *et al.* have shown that zinc therapy improves sexual competence by increasing penile thrusting and prolonging ejaculatory latency without disturbing arousability and motivation of male rats. However, further studies on pigs and monkeys are needed to evaluate the possible therapeutic use of zinc in sexual dysfunction.

In an era where the COH program is being optimized in all respects, a study by Thankam Verma and her team attempting to relate the antral follicle size on day 3 of downregulation to the in vitro fertilization (IVF) outcome will perhaps add a parameter to predict IVF outcome.

Case reports on successful pregnancies and a live birth after intracytoplasmic sperm injection in globozoospermia, late onset ovarian hyperstimulation with bilateral pleural effusion, and Herlyn Werner-Wunderlich syndrome also make for interesting reading.

Finally I want to draw your attention to a new initiative in ISAR. As you all are aware, ISAR has been publishing the National ART Registry of India (NARI) for the last 8 years. This was a retrospective analysis of ART cycles carried out in India. With advances in technology and to ensure constant availability of information, from this year onward, ISAR will be making this an online web-based registry. On behalf of ISAR and Dr. Manish Banker who has commendably been handling NARI since its inception, I request all of you to join this by submitting your data online. We look forward to your favorable response to help NARI go the electronic way and become e-NARI.

